# The federal plan for health science and technology’s response to the opioid crisis: understanding sex and gender differences as part of the solution is overlooked

**DOI:** 10.1186/s13293-018-0215-5

**Published:** 2019-01-07

**Authors:** Jill B. Becker, Carolyn M. Mazure

**Affiliations:** 1 Department of Psychology and the Molecular & Behavioral Neuroscience Institute, University of Michigan, 205 Zina Pitcher Place, Ann Arbor, MI 48109 USA; 2 Department of Psychiatry and Women’s Health Research at Yale, Yale University School of Medicine, 135 College Street, Suite, New Haven, CT 220 USA

**Keywords:** Opioid crisis, Sex differences, Gender-based strategies

## Abstract

The Fast-Track Action Committee on (the) Health Science and Technology Response to the Opioid Crisis recently released their draft report for public comment. This report provides the “roadmap” for a coordinated federal research and development response to the opioid crisis. Other than noting the important concerns regarding maternal and neonatal exposure to opioids, the report overlooks the laboratory, clinical, and epidemiological data that inform the need for further research on sex and gender differences in opioid addiction that have critical gender-based treatment and prevention implications. As we embark on research and development, investigations into the neurobiology of pain, opioid use, and addiction must include both females and males in model systems and, similarly, psychological and sociocultural investigations must study women and men. All data should be reported by sex and gender so that gender-specific treatment and prevention strategies derived from this research are provided to practitioners and the public. We encourage biomedical researchers and clinical care providers, as well as the public, to insist that a successful response to the opioid crisis should highlight the importance of understanding sex and gender differences in the current opioid epidemic.

## Background

In October 2018, the Fast-Track Action Committee (FTAC) on (the) Health Science and Technology Response to the Opioid Crisis released their draft report for public comment. The FTAC committee consisted of staff from United States governmental units including the National Institutes of Health, National Science Foundation, Center for Disease Control, Food and Drug Administration, Office of Science and Technology Policy, Department of Defense, United States Department of Agriculture, and other federal agencies impacted by the opioid crisis. According to the report (https://www.nih.gov/draft-ftac), “The White House National Science and Technology Council chartered the Opioid FTAC to support the President’s response to the opioid crisis by identifying (1) Research & Development (R&D) critical to addressing key gaps in knowledge and tools, and (2) opportunities to improve coordination of Federal R&D essential to combating the opioid crisis.”

This is a critically important report because it provides the guidance for the federal response to the opioid crisis. However, essential laboratory, clinical, and epidemiological data on sex and gender differences in opioid addiction are overlooked [1]. We assert that the national response should endorse and encourage sex and gender difference research and generate coordinated gender-based interventions that can more fully address the opioid epidemic.

As the report documents, the number of prescription overdose deaths dramatically increased between 1999 and 2016. What the report fails to mention is that women are more likely than men to be prescribed and use opioid analgesics [2]. And, as importantly, there are gender-specific risk factors for addiction and death from opioid overdoses [3, 4]. Moreover, over the past 50 years, the number of men who are addicted to heroin has been steadily decreasing, while the number of women who are addicted has been increasing such that the rates for women and men are now similar [2, 5].

## Main text

The biology of pain and opioid addiction is different for females and males. In research studies with rodents, most studies on pain have been conducted in males. When research includes female subjects, sex differences in the neural systems mediating the responses to pain- and opioid-related pain reduction are found [6]. There also are sex differences in the pathways conducting pain information, the mechanisms through which pain activates these pathways, the influence of gonadal hormones on opioid receptor expression, and opioid metabolism—all of which contribute to opioid-mediated pain reduction [6]. In summary, these findings show that females have an attenuated reduction of pain in response to opioids relative to males. This result is mirrored in clinical research where women have decreased pain reduction with opioids relative to men [6].

Clinical studies show that for drugs of abuse, the progression from casual use to addiction occurs more rapidly for women than men [7, 8]. Women also experience more negative adverse effects during withdrawal and are more likely than men to relapse [9]. In preclinical laboratory experiments, female rodents start self-administering opioids more rapidly than do males, and females find opioids more rewarding than males [7, 9]. As reviewed in [9], sex differences in the initial acquisition and intake of opioids are not due to metabolic differences, but appear to be related to sex differences in opioid signaling in the brain. Thus, there are sex differences in the neurological mechanisms mediating the response to opioids that underlie sex differences in addiction and increase addiction liability in females relative to males.

The report also advocates the study of “non-biological” contributions to opioid addiction (quotes added; pg. 5). In fact, women with opioid use disorder (OUD) are more likely than men with OUD to have experienced early trauma, been diagnosed with co-morbid depressive and anxiety disorders, and reported using opioids to manage stress as well as pain [10, 11]. Women with OUD also have greater functional impairment, which affects the capacity to obtain and retain employment and maintain stable housing (10.1016/j.cpr.2017.10.012; [10, 12]). Because most family caregivers are women, such impairment has a greater adverse effect on children and families. In addition, neonatal abstinence syndrome is on the rise as opioid use and addiction increases in women. Treatment for substance use has historically engendered greater stigma for women than men; thus, women have had greater reluctance to seek treatment. Women also report concern regarding losing custody of their children if they are identified as abusing substances. As a consequence, programs that include women-oriented services such as child care and domestic counseling tend to show better attendance and outcomes for women [13].

Furthermore, it should be pointed out that any adverse event resulting in a change in behavior, such as abusing opioids, induces changes in the brain. Consequently, while the original etiology of the contribution may be “non-biological,” adverse events have very real and substantial biological consequences. For example, childhood trauma, sexual abuse, or witnessing violence all cause changes in the brain that contribute to an increased risk for opioid addiction.

## Conclusions

Women suffer chronic pain and disability at greater rates than men [14, 15], are more likely to be prescribed opioids, and can become addicted more rapidly than men. The consequences of addiction are also different for women and men, with women showing a greater withdrawal response, more sporadic relapse than men, and different psychosocial outcomes. We conclude that sex and gender differences result from the interaction of biological, psychological, and sociocultural influences, and consideration of these differences in treatment and prevention strategies is fundamental to understanding the causes of and finding solutions to the opioid crisis.

Yet, there is considerable work to be done to understand how women and men differ in the biology, chemistry, and experience of pain and distress that precipitates misuse of and addiction to opioids. Our experimental models will not begin to yield the desired information until they employ appropriate models that include both females and males, and our clinical and epidemiological investigations will not uncover needed data until both women and men are studied. A successful response to the opioid crisis will only be found when scientists, practitioners, and the public incorporate the essential importance of understanding sex and gender differences into the solution for OUD.

## Abbreviations

FTAC: Fast-Track Action Committee
OUD: Opioid use disorder
R&D: Research & Development
